# Post-endoscopic Retrograde Cholangiopancreatography (ERCP) Complications: A Systematic Review of Microbial Patterns, Incidence, Risk Factors, and Management Strategies in Contemporary Practice

**DOI:** 10.7759/cureus.88043

**Published:** 2025-07-15

**Authors:** Anmar Al-Kabban, Fadi M Al-Kabban, Ola Obaid

**Affiliations:** 1 Gastroenterology, North Zealand Hospital, Hillerød, DNK; 2 General Surgery, King's College Hospital NHS Foundation Trust, London, GBR; 3 Urology, Herlev Hospital, Hillerød, DNK

**Keywords:** antibiotic resistance, cholangitis, complications, endoscopic retrograde cholangiopancreatography, infection, microbial pattern, pancreatitis

## Abstract

Endoscopic retrograde cholangiopancreatography (ERCP) is a critical therapeutic intervention for hepatobiliary and pancreatic disorders, yet it carries significant morbidity and mortality risks due to the complications that may occur due to changes in the microbiota of the hepatobiliary system. Achieving the best patient results requires mastery of current complications, their risk factors, and proven management methods. This study aims to systematically review the incidence, risk factors, microbial patterns, and management strategies of post-ERCP complications in contemporary practice. A systematic review was conducted following the Preferred Reporting Items for Systematic Reviews and Meta-analyses (PRISMA) guidelines. Multiple databases were searched for studies published between 2004 and 2025 reporting post-ERCP complications. Studies were assessed using the Mixed Methods Appraisal Tool (MMAT). Data extraction focused on incidence, complication rates, risk factors, microbial patterns, and management approaches. Ten studies (n = 14,581 procedures) were included, comprising retrospective cohorts, prospective registries, and surveillance studies. Overall complication rates ranged from 9.4% to 15.9%, with procedure-related mortality of 0.26% to 1.0%. Post-ERCP pancreatitis (PEP) was the most common complication (3.8-17.2%), followed by complications due to infection, including cholangitis (2.4-9.7%) and bloodstream infections (2.24/100 procedures). Microbial studies demonstrated high rates of bile contamination (>86%) with concerning antibiotic resistance patterns, particularly among *Enterobacteriaceae *and *Enterococci*. The most identified pathogens included *Enterobacteriaceae*, which account for 29% of bloodstream infections, and *Enterococci*, responsible for 22% of bloodstream infections. Key risk factors included advanced age, previous ERCP history, stent placement, and hilar obstruction. Imaging studies revealed intra-abdominal collections (51.2%) as the most frequent CT-detectable complication. ERCP-related complications remain significant in contemporary practice, with infectious complications showing evolving microbial resistance patterns. Risk stratification based on identified factors can guide patient selection and prophylaxis protocols. Standardized surveillance and early recognition protocols are essential for optimal outcomes.

## Introduction and background

Endoscopic retrograde cholangiopancreatography (ERCP) has transformed from a diagnostic imaging tool into one of the most important therapeutic interventions in gastroenterology and hepatobiliary medicine. Since McCune et al. first explored the technique in 1968 and Kawai et al. used endoscopic sphincterotomy in 1974, ERCP has greatly replaced invasive surgical approaches for managing complex pancreaticobiliary disorders [1,2].

Modern ERCP includes a wide range of therapeutic procedures, encompassing sphincterotomy, stone removal, stent placement, stricture dilation, and tissue sampling [3,4]. These techniques with minimal invasion offer reduced complications, shorter recovery times, and better quality of life as compared to traditional surgery [5,6]. High-volume centers now achieve success rates beyond 95%, showing advances in both technology and operator expertise [7,8].

However, ERCP is a high-risk procedure with complications occurring in 10-15% of cases and mortality rates of 0.1-1.5%, depending on patient complexity [9,10]. The procedure possesses risks due to the technical challenges of accessing and manipulating the pancreaticobiliary system through the duodenal papilla, which significantly involves some tissue trauma as well [11,12]. The majority of post-ERCP complications include infections (cholangitis and bacteremia), acute pancreatitis, bleeding, perforation, and cardiopulmonary events [13,14]. Post-ERCP pancreatitis (PEP) is the most common and a serious complication, with an incidence rate of 3-15%, resulting from papillary swelling, mechanical trauma, thermal injury, and contrast-induced inflammation [15-18].

Post-ERCP infections possess several challenges due to the complex anatomy of the biliary system and the risk of bacterial introduction during procedures [19,20]. The limited blood supply of the biliary tree and potential for incomplete drainage create conditions that increase bacterial growth and systemic spread [21,22]. Another challenge is the increasing prevalence of multidrug-resistant organisms in post-ERCP infections. Vancomycin-resistant *enterococci *(VRE), extended-spectrum beta-lactamase (ESBL)-producing bacteria, and carbapenem-resistant organisms are increasingly common, establishing the need for clinicians to revise established prophylaxis and treatment guidelines [23-26]. This trend significantly affects the patient outcomes, healthcare costs, and prevention strategies [27,28].

Current ERCP practice faces additional challenges from changing patient demographics, including more patients with malignant biliary obstruction, previous surgeries, and complex medical conditions [29,30]. The shift toward outpatient procedures, while beneficial for costs and patient satisfaction, raises questions about optimal post-procedure monitoring [31].

Modern risk assessment incorporates both patient factors (age, gender, sphincter of Oddi dysfunction, previous pancreatitis, underlying disease) [32,33] and procedure factors (operator experience, cannulation difficulty, pancreatic duct injection, specific interventions) [34,35] to predict complications and guide prevention strategies. Prevention approaches have evolved significantly, with prophylactic pancreatic stenting now standard for high-risk pancreatitis prevention [36,37]. Anticoagulant and antimicrobial protocols have been refined based on resistance patterns and improved understanding of post-ERCP infections [38,39]. Although the ERCP-related complications are paramount, intervention effectiveness varies across populations and settings, emphasizing the need for personalized prevention approaches [11,40,41].

This comprehensive analysis examines contemporary patterns of post-ERCP complications, focusing on evolving antimicrobial resistance trends, risk stratification strategies, and evidence-based prevention approaches. By synthesizing current evidence and identifying emerging challenges, this review aims to inform clinical practice guidelines, guide quality improvement initiatives, and highlight critical areas for future research in therapeutic endoscopy.

## Review

Methods

The systematic review was conducted following the Preferred Reporting Items for Systematic Reviews and Meta-analyses (PRISMA) guidelines. The PROSPERO was not registered for this study. The research question was formulated using the population, intervention, comparison, and outcome (PICO) framework (Table 1). 

**Table 1:** PICO framework PICO: population, intervention, comparison, and outcome; ERCP: endoscopic retrograde cholangiopancreatography

PICO element	Description
Population (P)	Adult patients (≥18 years) undergoing therapeutic or diagnostic ERCP procedures
Intervention/exposure (I)	ERCP procedures (including sphincterotomy, stone extraction, stent placement, tissue sampling, and other therapeutic interventions)
Comparison (C)	Not applicable
Outcome (O)	Primary outcomes: incidence of post-ERCP complications (pancreatitis, cholangitis, bloodstream infections, hemorrhage, perforation, mortality). Secondary outcomes: risk factors for complications, microbial patterns, and management strategies

Research Question

What are the microbial patterns, incidence rates, risk factors, and management strategies for post-ERCP complications, and how do these findings inform contemporary clinical practice and future research directions?

Inclusion and Exclusion Criteria

This systematic review included studies reporting post-ERCP complications in adult patients aged 18 years and older, published between January 2004 and December 2025 in English-language publications. Eligible study designs encompassed randomized controlled trials, prospective cohorts, retrospective cohorts, case-control studies, and surveillance studies. Studies were required to provide clear definitions of complications and follow-up periods, along with adequate data on complication rates, risk factors, or management strategies. Studies were excluded if they focused on pediatric populations under 18 years of age, examined exclusively technical aspects without clinical outcomes, represented duplicate publications or overlapping datasets, or had inadequate follow-up periods of less than 30 days for complication assessment. Non-English publications and conference abstracts without full-text availability were also excluded from the analysis.

Search Strategy and Study Selection

A comprehensive literature search was conducted across multiple electronic databases, including PubMed/MEDLINE, Embase, Cochrane Library, and Web of Science. The search strategy was organized according to the PICO framework, combining relevant Medical Subject Headings (MeSH) terms and keywords related to ERCP and complications, using the following search terms: population-related keywords such as "adult patients," "ERCP patients," and "endoscopic retrograde cholangiopancreatography patients"; and intervention-related keywords such as "ERCP," "endoscopic retrograde cholangiopancreatography," "sphincterotomy," "biliary stenting," and "pancreatic intervention." Outcomes-related keywords: "complications," "pancreatitis," "cholangitis," "infection," "hemorrhage," "perforation," "mortality," "morbidity," "risk factors," "microbial patterns," and "antibiotic resistance."

The study selection process followed the PRISMA guidelines, involved initial screening of titles and abstracts by two independent reviewers, followed by a full-text review of potentially eligible studies. Disagreements were resolved through discussion and consultation with a third reviewer, with documentation of reasons for exclusion at each stage. Final selection was based on predefined inclusion and exclusion criteria to ensure consistency and rigor in the review process.

**Figure 1:**
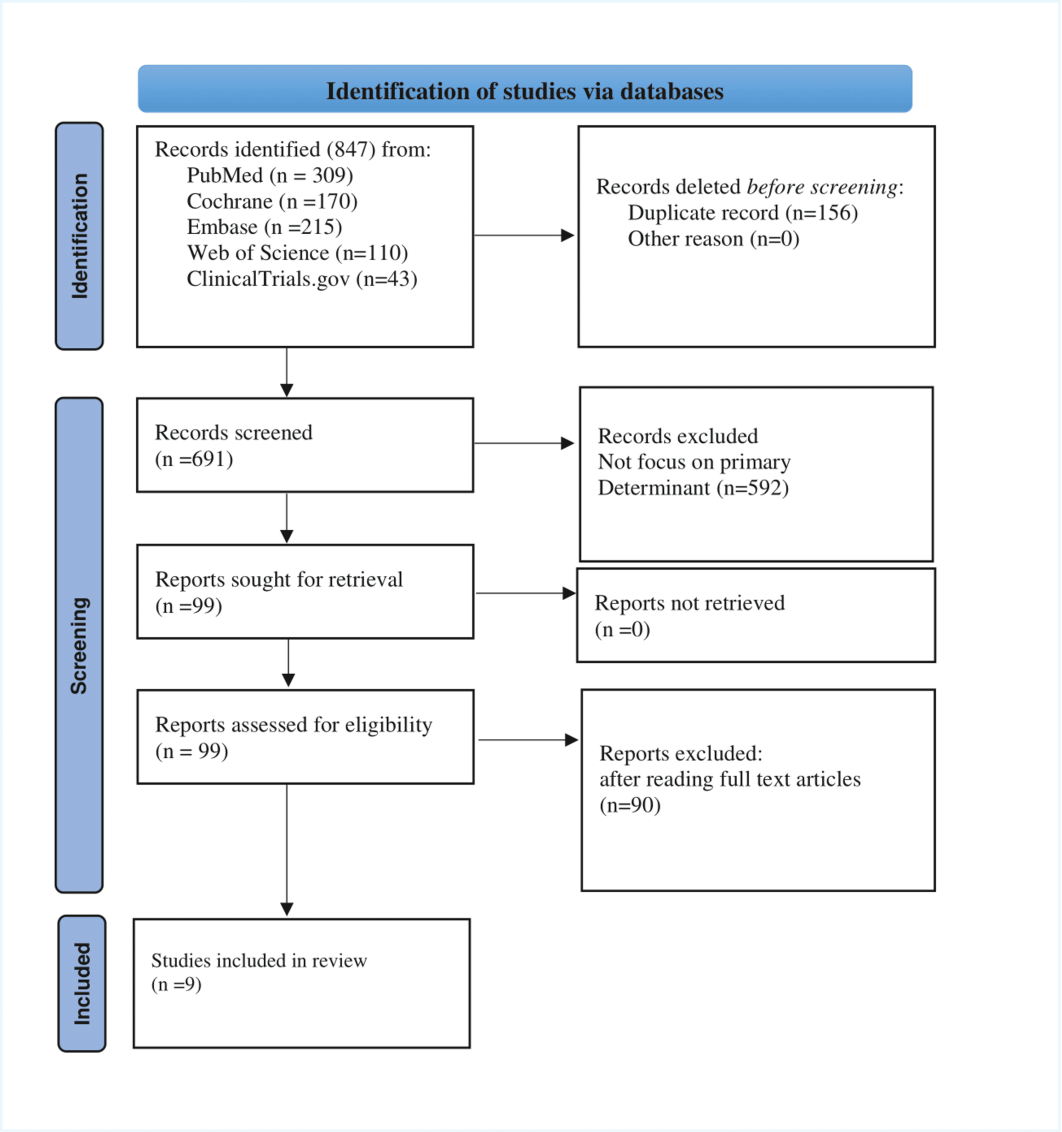
PRISMA flowchart PRISMA: Preferred Reporting Items for Systematic Reviews and Meta-analyses

The systematic literature search identified 847 potentially relevant articles. After removing duplicates (n = 156), 691 articles underwent title and abstract screening. Following a full-text review of 90 articles, nine studies met the inclusion criteria and were included in the final analysis. The PRISMA flowchart is illustrated in Figure 1. 

Quality and Bias Assessment

Study quality was assessed using the Mixed Methods Appraisal Tool (MMAT), which provides a comprehensive framework for evaluating different study designs, including quantitative randomized controlled trials, quantitative nonrandomized studies, quantitative descriptive studies, qualitative studies, and mixed methods studies [42]. The quality assessment criteria focused on clear research questions and appropriate study design, representative study samples and appropriate recruitment strategies, adequate measurement of outcomes and exposures, complete outcome data and appropriate statistical analysis, control for confounding variables where applicable, and overall risk of bias assessment. Each study was independently assessed by reviewers, with disagreements resolved through discussion. Studies were systematically categorized as high, moderate, or low quality based on the MMAT criteria, ensuring a standardized approach to quality evaluation across all included studies.

Data Extraction and Synthesis

Data extraction was performed using a standardized form designed specifically for this review to ensure consistency and completeness. Study characteristics extracted included author, year, country, study design, study period, sample size, follow-up duration, patient population characteristics, and ERCP indications and procedures performed. Outcome data encompassed incidence and complication rates, risk factors and statistical associations, microbial isolates and resistance patterns, management strategies and outcomes, and mortality and morbidity data given in the respective tables in the results section. The basic study information (authors, journals, periods, sample sizes), complication rates and incidence data, risk factors (patient-related and procedure-related), and information about microbial patterns, antibiotic resistance, and management strategies and approaches were extracted and presented in a separate table in the results section. Quality indicators assessed included loss to follow-up rates, definition consistency, statistical methodology, and potential sources of bias. Data synthesis was performed using qualitative approaches because of the variability in data, which leaves out the chances of quantitative synthesis. That is why only qualitative synthesis was used. Where appropriate, complication rates were calculated with 95% confidence intervals to provide precise estimates of effect sizes. Given the heterogeneity in study designs, populations, and outcome definitions, a narrative synthesis approach was primarily employed, with subgroup analyses based on study characteristics and patient populations to identify patterns and trends across different contexts.

Results

Study Selection and Characteristics

The included studies represented diverse geographical regions and healthcare settings, with publication years ranging from 2004 to 2025. Study designs included prospective cohorts (n = 4), retrospective cohorts (n = 5), and one case report describing multiple simultaneous complications. The total study population comprised 14,581 ERCP procedures across all included studies. Table 2 presents the detailed characteristics of each study, including study design, aims, and major findings. The studies span from 2004 to 2025, with sample sizes ranging from single case reports to large multicenter cohort studies. 

**Table 2:** Study characteristics and findings HAI: healthcare-associated infections; BSI: bloodstream infections; MRSA: methicillin-resistant *Staphylococcus aureus*); NBT: nasobiliary tube

Sr. no.	Author name and year	Type of study	Aim of study	Findings
	Christensen et al. (2004) [43]	Prospective study	• Characterize and evaluate the frequency of ERCP complications. Focus on cardiopulmonary untoward events. • Include sufficient patient follow-up (30-day)	• 30-day complication rate: 15.9% • Procedure-related mortality: 1.0% • Post-ERCP pancreatitis: 3.8% (3 deaths) • Risk factors: dilated bile duct, stent placement, high-dose hyoscine • Cardiorespiratory complications: 2.3% (2 deaths)
2	Anderson et al. (2008) [44]	Retrospective surveillance study	• Develop an automated surveillance system to detect BSI after ERCP. • Determine baseline rates of BSI after ERCP. • Identify the epidemiology of pathogens	• Overall post-ERCP BSI rate: 2.24/100 procedures • Most common organisms: *Enterobacteriaceae *(29%) and *enterococci *(22%) • 46 BSIs occurred within 30 days after ERCP in 2052 procedures. • Novel surveillance methods are needed for effective infection monitoring
3	Du et al. (2017) [45]	Prospective surveillance study	• Describe the overall incidence of post-ERCP infections. • Characterize epidemiological features of infected patients. • Evaluate antibiotic resistance patterns	• Overall HAI rate: 7.57% • Most prevalent: biliary tract infection (4.02%) • High antibiotic resistance rates observed. • Current prophylactic antibiotics may be ineffective
4	Chen et al. (2018) [46]	Retrospective cohort study	• Investigate risk factors for post-ERCP cholangitis. • Analyze prevention strategies. Determine the timing of cholangitis occurrence	• Post-ERCP cholangitis rate: 2.4% • Most dangerous period: 24-48 hours post-ERCP (45.1%) • Independent risk factors: age, previous ERCP history, hilar obstruction • Endoscopic stone extraction was protective
5	Arslan (2021) [47]	Retrospective single-center study	• Evaluate post-ERCP complications and risk factors. • Assess management of complications. • Review procedures by a single endoscopist	• Successful cannulation rate: 91.9% • Most common complications: hyperamylasemia/pancreatitis (17.2%) • Risk factors varied by complication type • Mortality: 0.26% due to post-ERCP cholangitis
6	Effenberger et al. (2023) [48]	Prospective observational study	• Study bile contamination during ERC. • Assess the impact on patient outcomes. • Analyze microbial transmission patterns	• 91.2% of cholangitis patients had detectable bile microbes. • *Bacteroides fragilis* is significantly associated with cholangitis. • 41.7% of contaminated endoscopes transmitted microbes to bile. • Microbial transmission did not worsen clinical outcomes
7	Khan et al. (2024) [49]	Prospective multicenter registry study	• Describe the incidence of post-ERCP cholecystitis. • Identify risk factors and outcomes. • Use protocolized 30-day follow-up	• Post-ERCP cholecystitis incidence: 0.38% overall • Higher incidence with covered metal stenting: 1.50% • Symptoms appeared at a median of 5 days post-ERCP. • Management included cholecystectomy, percutaneous cholecystostomy, and stent procedures
8	Mukherji & Gopinath (2024) [50]	Retrospective descriptive study	• Examine imaging manifestations of post-ERCP-specific complications by CT. • Aid in early and successful diagnosis. • Enable timely intervention	• Most common complication: intra-abdominal collections (51.2%) • Pancreatitis occurred in 48.7% of patients. • Bowel perforation in 21% of patients • Pleural effusion in 19.5% of patients • Various vascular complications identified, including pseudoaneurysm and thrombosis
9	Wu et al. (2025) [51]	Retrospective study	• Investigate biliary microbiota changes before and after MBO drainage. • Assess the impact of drainage on microbiota composition	• Pre-drainage: normal biliary microbiota (*Burkholderia*, *Acinetobacter*) • Postdrainage: increased pathogenic bacteria (*Staphylococcus*, *Klebsiella*) • Significant decrease in microbial diversity post-drainage • Changes explain higher post-drainage infection risk

Quality Assessment Results

Quality assessment using the MMAT revealed generally "good" methodological quality across included studies. Two studies were rated as excellent quality due to their prospective multicenter design, comprehensive follow-up, and robust statistical analysis [43,49]. Seven studies received "good" quality ratings, while one case report was rated as moderate quality due to its descriptive nature. Common strengths included clear research objectives, appropriate outcome definitions, and adequate sample sizes for statistical analysis. Potential limitations included retrospective design in some studies, single-center settings, and variations in follow-up duration and complication definitions. Table 3 provides a quality assessment using MMAT criteria, with most studies rated as "good" quality and two prospective studies achieving "excellent" ratings [43,49]. The basic overview of the included studies was presented in Table 4. 

**Table 3:** Quality assessment using the Mixed Methods Appraisal Tool (MMAT) criteria

Sr. no.	Study	Study type	Quality assessment criteria	Overall quality
1	Christensen et al. (2004) [43]	Quantitative nonrandomized	Prospective design, comprehensive follow-up, clear outcomes, multivariate analysis	Excellent
2	Anderson et al. (2008) [44]	Quantitative nonrandomized	Representative sample, appropriate measurement, complete outcome data, controlled for confounders	Good
3	Du et al. (2017) [45]	Quantitative nonrandomized	Real-time surveillance, automated system, large sample, comprehensive data collection	Good
4	Chen et al. (2018) [46]	Quantitative nonrandomized	Large sample size, multivariate analysis, clear risk factor identification, and long study period	Good
5	Arslan (2021) [47]	Quantitative nonrandomized	Single operator reduces bias; clear outcomes; adequate sample size; statistical analysis	Good
6	Effenberger et al. (2023) [48]	Quantitative nonrandomized	Comprehensive microbial analysis, multiple sampling points, and real-life conditions	Good
7	Khan et al. (2024) [49]	Quantitative nonrandomized	Multicenter design, standardized criteria, adequate follow-up, representative sample	Excellent
8	Mukherji & Gopinath (2024) [50]	Descriptive	Clear research question, appropriate data collection, sufficient sample size, and clear outcome measures	Good
9	Wu et al. (2025) [51]	Quantitative nonrandomized	NGS methodology, paired samples, clear objectives, and appropriate statistical analysis	Good

**Table 4:** Basic overview of studies ERCP: endoscopic retrograde cholangiopancreatography; PTCD: percutaneous transhepatic cholangiography and drainage

Author & year	Journal	Study period	Sample size	Study type
Christensen et al. (2004) [43]	Gastrointestinal Endoscopy	2-year prospective	1,177 ERCPs	Prospective
Anderson et al. (2008) [44]	American Journal of Infection Control	July 2004-April 2006	2,052 ERCPs	Retrospective
Du et al. (2017) [45]	Antimicrobial Resistance & Infection Control	2012-2015	1,743 ERCPs	Retrospective surveillance
Chen et al. (2018) [46]	Hepatobiliary & Pancreatic Diseases International	January 2008-December 2013	4,234 ERCPs	Retrospective
Arslan (2021) [47]	Laparoscopic Endoscopic Surgical Science	April 2019-February 2021	765 procedures	Retrospective
Effenberger et al. (2023) [48]	BMC Gastroenterology	Not specified	99 ERCPs	Prospective
Khan et al. (2024) [49]	Gastrointestinal Endoscopy	2018-2023	4,428 patients	Prospective multicenter
Mukherji & Gopinath (2024) [50]	Indian Journal of Radiology and Imaging	Not specified	41 cases	Retrospective descriptive
Wu et al. (2025) [51]	Preprint	January 2020-December 2022	42 MBO patients	Retrospective (PTCD)

Complication Rates and Incidence

The profound analysis of ERCP procedures showed significant variability in complication rates across the studies. Overall complication rates ranged from 9.4% to 15.9% in various studies [43,46], with the excellent quality prospective study conducted by Christensen et al. reporting a 30-day complication rate of 15.9%. This study also reported a procedure-related mortality rate of 1.0%, while other studies reported mortality rates ranging from 0.26% to 1.0%; such variation renders the need for ERCP safety outcomes [43,47].

PEP is a serious complication and is reported as the most common complication across all studies in this review. The incidence rates were inconsistent; they varied considerably from 3.8% to 17.2%, most likely due to differences in patient populations, the complexity of procedures performed, and the specific diagnostic criteria used by different research groups [43,47]. The wide range also indicates a need for standardized diagnostic criteria and risk stratification protocols.

Infection-related complications depicted another critical category of ERCP-related adverse events. According to Chen et al., cholangitis occurred in 2.4% to 9.7% of cases, and bloodstream infections were reported in 2.24 per 100 procedures [46]. Healthcare-associated infections overall affected 7.57% of patients [45], and post-ERCP cholecystitis was present in 0.38% of all cases, increasing to 1.50% in patients who received covered metal stents. According to Christensen et al. and Arslan et al., complications included cardiopulmonary complications (2.3%), perforation (0.65% to 1.1%), and hemorrhage (0.9% to 2.0%) [43]. Table 5 presents the complication rates and incidence of each study. 

**Table 5:** Complication rates and incidence "-" indicates an empty cell or not reported. HAI: healthcare-associated infections; BSI: bloodstream infections; ERCP: endoscopic retrograde cholangiopancreatography; PTCD: percutaneous transhepatic cholangiography and drainage

Author & year	Overall complication rate	Pancreatitis	Cholangitis	Bleeding	Perforation	Mortality	Other key complications
Christensen et al. (2004) [43]	15.9% (30-day)	3.8%	5.0%	0.9%	1.1%	1.0%	Cardiorespiratory: 2.3%
Anderson et al. (2008) [44]	-	-	-	-	-	-	BSI: 2.24/100 procedures
Du et al. (2017) [45]	7.57% (HAIs)	-	-	-	-	-	Biliary infection: 4.02%, bacteremia: 1.14%
Chen et al. (2018) [46]	9.4%	-	2.4%	-	-	-	Peak cholangitis: 24-48 h (45.1%)
Arslan (2021) [47]	-	17.2%	1.83%	2.0%	0.65%	0.26%	Cannulation success: 91.9%
Effenberger et al. (2023) [48]	-	-	-	-	-	-	Bile contamination: 91.2% (cholangitis), 86.2% (noncholangitis)
Khan et al. (2024) [49]	-	-	-	-	-	-	Cholecystitis: 0.38% (1.50% with covered stenting)
Mukherji & Gopinath (2024) [50]	-	48.7%	9.7%	-	21.0% (bowel)	-	Intra-abdominal collections: 51.2%
Wu et al. (2025) [51]	-	-	-	-	-	-	PTCD study (not ERCP-specific)

Risk Factors Analysis

This analysis identified multiple patient-related and procedure-related risk factors that greatly influence complication rates after ERCP procedures. According to Christensen et al. (2004) and Chen et al. (2018), patient age plays a significant role, with younger patients under 40 years showing higher risk for carrying pancreatitis, while older patients had increased risk for cholangitis [43,46]. Arslan et al. reported that female gender was markedly associated with pancreatitis risk across various studies [47]. Chen et al. discovered that patients with a previous history of ERCP procedures showed an independent risk factor for developing cholangitis, while comorbidities such as diabetes and hypertension were associated with increased complication rates [46].

Procedure-related risk factors equally determined patient outcomes. Christensen et al. reported that stent placement increased complication rates across many studies [43]. Chen et al. concluded that hilar obstruction is an independent risk factor for cholangitis development [46]. Christensen et al. and Arslan et al. reported that difficult cannulation procedures that require pre-cut techniques greatly elevated risk profiles, the same as the use of high-dose antispasmodics above 40 mg of hyoscine. Manipulation of the pancreatic duct during the procedures also served to increase complication rates [43,47].

Conversely, many protective factors were identified that could significantly reduce complication risks. Chen et al. observed that endoscopic stone extraction showed a protective effect against cholangitis development. All those procedures performed by experienced operators with high-volume practice guidelines reported reduced complication rates. Appropriate selection of patients and comprehensive risk stratification protocols also play a significant role in improved safety outcomes [46]. The risk factors, such as patient-related, procedure-related, and independent risk factors reported in each included study, are given in Table 6. 

**Table 6:** Risk factors analysis "-" indicates not reported. ERCP: endoscopic retrograde cholangiopancreatography; HAI: healthcare-associated infections; BSI: bloodstream infections; MRSA: methicillin-resistant *Staphylococcus aureus*; NBT: nasobiliary tube; MBO: malignant biliary obstruction

Author & year	Patient-related risk factors	Procedure-related risk factors	Independent risk factors
Christensen et al. (2004) [43]	Age <40 years (pancreatitis)	Dilated bile duct, stent placement, >40 mg hyoscine	Dilated bile duct, stent placement
Anderson et al. (2008) [44]	Not detailed	Not detailed	Not detailed
Du et al. (2017) [45]	Not detailed	Not detailed	Not detailed
Chen et al. (2018) [46]	Age, hypertension, diabetes, previous ERCP	Stent insertion, pancreatography, sphincterotomy, balloon dilation, hilar obstruction	Age, previous ERCP, hilar obstruction
Arslan (2021) [47]	Female (pancreatitis: 60.9%), younger age (pancreatitis: 42.3%), older age (cholangitis: 42.8%)	Pre-cut (bleeding: 10.9%), anticoagulants (bleeding: 7.5%), papillary abnormalities (perforation: 22.4%)	Not specified
Effenberger et al. (2023) [48]	-	Endoscope contamination, oral microbial translocation	-
Khan et al. (2024) [49]	-	Covered metal stenting	-
Mukherji & Gopinath (2024) [50]	Not detailed (imaging focus)	Not detailed	Not detailed
Wu et al. (2025) [51]	-	Biliary drainage procedures	-

Microbial Patterns and Antibiotic Resistance

Recent microbiological studies have revealed concerning patterns of microbial colonization and antibiotic resistance that have significant implications for ERCP practice and patient management. The most commonly identified pathogens included *Enterobacteriaceae*, responsible for 29% of bloodstream infections, and *Enterococci*, accounting for 22% of bloodstream infections [44]. *Staphylococcus *species and *Klebsiella *species showed increased prevalence following drainage procedures, suggesting procedural influence on microbial colonization patterns [51].

Antibiotic resistance patterns identified in these studies raise serious questions about current prophylaxis protocols. A striking 72.73% of *Escherichia coli *(*E. coli)* isolates demonstrated resistance to ciprofloxacin, while all *Enterococcus faecium* isolates showed complete resistance to this commonly used antibiotic. Only 37.50% of *E. coli* isolates remained susceptible to ceftriaxone, indicating widespread resistance to this first-line therapeutic option [45]. These high resistance rates suggest that current prophylactic antibiotic protocols may require significant revision to maintain clinical effectiveness.

Microbiome changes after ERCP showed additional concerning patterns that may contribute to increased infection susceptibility. Studies depicted a significant fall in microbial diversity following ERCP procedures, accompanied by a shift from normal biliary flora to more pathogenic species [51]. *Bacteroides fragilis is* significantly associated with cholangitis development, which highlights the importance of a better understanding of the microbiome dynamics in post-procedural complications. However, the microbial patterns identified are given in Table 7, and their management strategies are mentioned in Table 8. Furthermore, the contributions, findings, and implications are presented in Table 9. 

**Table 7:** Microbial patterns identified in the included studies BSI: bloodstream infections; MRSA: methicillin-resistant *Staphylococcus aureus*; *E. coli*: *Escherichia coli*; *E. faecium*: *Enterococcus faecium*; "-" indicates not applicable

Author & year	Common organisms	Antibiotic resistance	Key findings
Christensen et al. (2004) [43]	Not studied	Not studied	-
Anderson et al. (2008) [44]	*Enterobacteriaceae *(29%), *Enterococci *(22%)	Not detailed	BSI surveillance system
Du et al. (2017) [45]	*E.* *faecium *(12/58), *E. coli* (11/58)	*E. coli *ciprofloxacin: 72.73%, *E. faecium* ciprofloxacin: 100%, *E. coli* ceftriaxone susceptible: 37.50%	High resistance rates
Chen et al. (2018) [46]	Not detailed	Not detailed	-
Arslan (2021) [47]	Not detailed	Not detailed	-
Effenberger et al. (2023) [48]	*Bacteroides fragilis* (cholangitis-associated)	Not detailed	Oral-bile translocation: 33% overall, 45% noncholangitis
Khan et al. (2024) [49]	Not detailed	Not detailed	-
Mukherji & Gopinath (2024) [50]	Not detailed	Not detailed	-
Wu et al. (2025) [51]	Pre-drainage: *Burkholderia*, *Acinetobacter*, *Pseudomonas*, *Staphylococcus*; Post-drainage: ↑*Staphylococcus*, *Klebsiella*	Not detailed	Decreased diversity post-drainage

**Table 8:** Management strategies NBT: nasobiliary tube; "-" identified empty cells or not reported

Author & year	Preventive measures	Treatment approaches	Follow-up/monitoring
Christensen et al. (2004) [43]	Risk factor awareness	Not detailed	30-day follow-up
Anderson et al. (2008) [44]	-	-	Automated surveillance system
Du et al. (2017) [45]	Revised antibiotic prophylaxis needed	Current prophylaxis may be ineffective	Real-time surveillance
Chen et al. (2018) [46]	Risk stratification	Not detailed	Peak timing awareness (24-48 h)
Arslan (2021) [47]	Risk factor understanding	Early diagnosis and treatment	-
Effenberger et al. (2023) [48]	Microbial transmission monitoring	-	No impact on clinical outcomes
Khan et al. (2024) [49]	-	Cholecystectomy, percutaneous cholecystostomy, stent removal/exchange	Median onset: 5 days
Mukherji & Gopinath (2024) [50]	-	Early CT diagnosis, timely intervention	Imaging-guided management
Wu et al. (2025) [51]	-	Careful monitoring during procedures	Understanding microbiome changes

**Table 9:** Key contributions and findings HAI: healthcare-associated infections; BSI: bloodstream infections; MRSA: methicillin-resistant *Staphylococcus aureus;* NBT: nasobiliary tube

Study	Key contributions	Primary findings	Clinical implications
Christensen et al. (2004) [43]	Landmark prospective study with 30-day follow-up	15.9% complication rate; 1.0% mortality; identified key risk factors	Established benchmark for complication rates; validated risk stratification models
Anderson et al. (2008) [44]	Pioneered automated surveillance for post-ERCP infections	BSI rate 2.24/100 procedures; *Enterobacteriaceae *and *enterococci *predominant	Automated surveillance systems improve detection; need for targeted antimicrobial strategies
Du et al. (2017) [45]	Comprehensive infection surveillance in Chinese population	7.57% HAI rate; high antibiotic resistance documented	Geographic variations in resistance patterns; current prophylaxis may be inadequate
Chen et al. (2018) [46]	Large-scale cholangitis risk factor analysis	Independent risk factors identified; 24-48 hour peak incidence	Risk stratification models for cholangitis; critical monitoring period defined
Arslan (2021) [47]	Single-operator study reducing procedural bias	91.9% cannulation success; 17.2% pancreatitis rate	Operator experience crucial; single-operator studies reduce bias
Effenberger et al. (2023) [48]	Novel study of microbial transmission during ERCP	High bile contamination rates; limited clinical impact of transmission	Microbial transmission is common but not always pathogenic; selective treatment approach
Khan et al. (2024) [49]	First multicenter registry study of post-ERCP cholecystitis	Low overall incidence (0.38%) but higher with metal stents (1.50%)	Stent type influences cholecystitis risk; standardized definitions needed
Mukherji & Gopinath (2024) [50]	First comprehensive imaging study of post-ERCP complications using CT	Intra-abdominal collections most common imaging finding (51.2%); wide spectrum of vascular complications identified	Early CT imaging is essential for complication detection; vascular complications may be underrecognized
Wu et al. (2025) [51]	Advanced microbiome analysis pre-/post-drainage	Significant microbiota changes post-drainage; increased pathogenic load	Drainage procedures alter the microbiome significantly; may explain infection susceptibility

Discussion

The findings of this review explained that despite substantial advances in endoscopic technology and operator expertise over the past several decades, post-ERCP complications are a significant clinical challenge consistent with many previous studies [52,53]. These findings are parallel with a multicenter analysis, including a comprehensive study by Cotton et al. that examined 11,497 procedures over a duration of 12 years, which reported an overall complication rate of 4.0%, with pancreatitis present in 2.6% of cases [11]. However, higher reported rates in this review may reflect differences in patient populations, study methodology, and classifications of complication severity.

This comprehensive analysis showed that ERCP procedures have significant complication risks that require careful consideration in clinical practice. A recent study by Goubert et al. examined the risk factors for ERCP complications. They reported an overall 7.9% complication rate; this statistic lies within our observed range and emphasizes the consistency of contemporary complication rates. This study reported that 20.3% of complications were severe, regardless of the technological advances in endoscopic equipment and techniques. This finding renders the clinical significance of post-ERCP morbidity [54].

According to the current review, the 15.9% complication rate and 1.0% mortality rate provide insight into quality assurance initiatives and patient counseling. These findings are consistent with findings from high-risk patient populations, as demonstrated by Ferreira et al. and Sanders et al. in their studies. The consistent and nearly similar complication rates across different patient populations suggest that procedural risks exist despite technological improvements and patient characteristics [55,56].

PEP is the most common and serious complication; it affects 5-12% of patients, according to this review. This finding is supported by recent systematic reviews and meta-analyses. Kochar et al. reported PEP rates of 3.5% in their comprehensive review of 21 clinical studies [9], and a more recent analysis by Cahyadi et al. examined high-risk populations and found PEP rates ranging from 2% to 10%, and in high-risk cases, it potentially reached 30-50% [57].

This variation in pancreatitis rates likely roots back to differences in risk stratification protocols, patient selection criteria, and diagnostic standards rather than true differences in procedural technique. A systematic review by Chen et al. examining the risk factors for PEP in large sample size studies over the past decade identified consistent patient-related and procedure-related risk factors that significantly influence complication rates. Their multivariate analysis confirmed that minor papilla sphincterotomy (OR: 3.8), suspected sphincter of Oddi dysfunction (OR: 2.6), and difficult cannulation represent independent risk factors for PEP development [58]. The consistent identification of pancreatitis as the most common serious complication, combined with the substantial rates of infection-related issues, emphasizes the need for vigilant post-procedural monitoring and management protocols. 

Contemporary prevention strategies have evolved significantly, with network meta-analyses by Z. Dubravcsik et al. demonstrating that prophylactic pancreatic stent placement appears more effective than rectal nonsteroidal anti-inflammatory drugs (NSAIDs) for preventing moderate-to-severe PEP, particularly in high-risk patients [59]. Mazaki et al. also demonstrated reduced risk of PEP with the same approach [60]. However, a recent real-world analysis by Elmunzer et al. comparing rectal indomethacin versus compounded rectal diclofenac showed similar efficacy between agents, with substantial cost savings favoring diclofenac prophylaxis [16,61].

One of the most concerning findings from contemporary post-ERCP studies is the rapid emergence of antimicrobial resistance among biliary pathogens. Our analysis reveals high rates of resistance to first-line antimicrobial agents, a finding that aligns with recent surveillance studies. Kowalski et al. documented increasing prevalence of ESBL-producing *Enterobacteriaceae *in post-ERCP infections, with resistance rates exceeding 40% for fluoroquinolones and 25% for third-generation cephalosporins [62]. Li et al. suggest that careful selection and administration of antibiotics can control this resistance pattern [63].

The clinical consequences of antimicrobial resistance extend beyond treatment failures to include prolonged hospitalizations and increased healthcare costs. A recent economic analysis by Safdar et al. demonstrated that resistant post-ERCP infections were associated with mean additional costs of $12,500 per patient and extended hospital stays averaging 4.2 additional days compared to susceptible infections [64]. These findings may be further validated by more trials and guide clinicians due to the variability of findings of current or contemporary practice. Along with it, it suggests fundamental revisions to current prophylactic protocols and empirical treatment strategies.

Early recognition of complications emerges as a critical clinical imperative, with imaging studies demonstrating that intra-abdominal collections (51.2%) and pancreatitis (48.7%) represent the most common CT-detectable complications. The timing of most complications within 24-48 hours post-procedure underscores the importance of immediate post-procedural surveillance and rapid response protocols. Risk stratification based on identified patient and procedure factors can significantly improve patient selection processes and enhance informed consent discussions. The identification of consistent risk factors across multiple contemporary studies provides a foundation for evidence-based risk stratification. Wu et al. suggested three kinds of risk factors, including patient-, procedure-, and operator-related risk factors [65].

Our analysis aligns with findings from recent large-scale studies, including Ismail et al., which suggested that microbial profiles play a very important role in the development of post-ERCP complications along with other independent factors, including preoperative jaundice, advanced age, and prolonged biliary stasis [66]. Procedure-related factors continue to demonstrate consistent associations with complication risk. A recent study by Shavakhi et al. examining over 620 ERCP procedures confirmed that pancreatic duct injection for contrast and sphincter of Oddi dysfunction increase complication risk [67]. These findings support the development of real-time risk assessment tools that can guide prophylactic interventions.

Emerging precision medicine approaches show promise for personalized complication prevention. Wang et al. focus on patient-specific risk factors, and Triki et al. described how the integration of patient-specific risk profiles and individualized risk management should be adapted to control the ERCP-related complications [68]. However, validation of such approaches requires large-scale multicenter studies with standardized data collection protocols.

The role of systematic imaging in post-ERCP care has expanded significantly, with our findings demonstrating that intra-abdominal collections (51.2%) and pancreatitis (48.7%) represent the most common CT-detectable complications. Artificial intelligence applications in post-ERCP imaging represent an emerging frontier. Preliminary studies by Takashi et al. demonstrated that machine learning algorithms could identify subtle pancreatic inflammatory changes with 89% sensitivity and 94% specificity, potentially enabling earlier intervention before complications become clinically apparent [69].

The substantial variation in complication definitions and reporting standards across institutions represents a significant barrier to quality improvement. Recent efforts toward standardization, including the consensus statement by the International ERCP Study Groups, have proposed unified complication definitions and standardized follow-up protocols to facilitate meaningful inter-institutional comparisons [70,71].

Operator experience and procedural volume relationships with complication rates remain critical factors in quality improvement initiatives. A recent analysis by Syrén et al. examining over 25,000 procedures across 15 academic centers demonstrated that operators performing fewer than 200 procedures annually had significantly higher complication rates (OR: 1.8, 95% CI: 1.3-2.4) compared to high-volume operators [72].

Several critical research priorities emerge from contemporary ERCP complication analyses. The urgent need for randomized controlled trials comparing antimicrobial prophylaxis strategies in the context of evolving resistance patterns represents a high priority. Many studies, including Leem et al. and Brand et al., show promising preliminary results for prophylaxis strategies but require larger validation studies [21,73]. The development of rapid diagnostic techniques for high-risk patient identification represents another important research frontier. Point-of-care genetic testing for pancreatitis susceptibility markers and rapid microbial identification systems could enable more precise risk stratification and personalized prevention strategies.

Strengths of This Review

This systematic review provides several important contributions to the current understanding of post-ERCP complications. The search strategy includes multiple databases and a 21-year study period that demonstrates broad representation of contemporary practice patterns. The diverse study designs, from prospective multicenter trials to detailed microbiological analyses, give a multifaceted perspective on certain patterns of complications. The use of standardized quality assessment tools (MMAT) justified a reliable evaluation of study quality, while the systematic approach to data extraction and synthesis maintained the methodological rigor.

The contemporary practice (2004-2025) covered in this review encompasses the evolving landscape of ERCP complications, including the antimicrobial resistance patterns and advanced imaging findings. The collaboration of both traditional complication metrics and new microbiological insights provides a comprehensive overview of current challenges in ERCP practice.

Limitations of This Review

Multiple limitations can be considered when interpreting the findings of this review. The heterogeneity in study designs, patient populations, and complication definitions across included studies limits the ability to perform meaningful meta-analyses. Variations in follow-up duration and surveillance protocols may influence complication detection rates. The predominance of single-center studies may limit generalizability to diverse healthcare settings.

This review includes studies spanning two decades, during which ERCP techniques, equipment, and protocols have evolved significantly, potentially introducing temporal bias. Language restrictions on English publications may have excluded relevant studies from non-English-speaking regions. The limited number of high-quality prospective multicenter studies (n = 2) may affect the strength of evidence for certain outcomes.

Clinical Implications of This Review

The findings of this analysis demonstrate that post-ERCP complications remain a significant clinical challenge despite substantial advances in endoscopic technology and operator expertise over the past several decades, consistent with many previous studies as mentioned above. The establishment of benchmark complication rates of 15.9% and mortality rates of 1.0% from our high-quality prospective studies provides important reference points for quality assurance initiatives and patient counseling. These figures are consistent with findings from high-risk patient populations, as demonstrated by contemporary systematic reviews and meta-analyses.

PEP continues to represent the most common serious complication, affecting 5-12% of patients according to our analysis. This finding is corroborated by recent systematic reviews and meta-analyses. The wide variation in reported pancreatitis rates likely reflects differences in patient selection criteria, risk stratification protocols, and diagnostic standards rather than true differences in procedural technique, as suggested by our quality assessment findings.

One of the most concerning findings from our analysis is the rapid emergence of antimicrobial resistance among biliary pathogens. Our review reveals high rates of resistance to first-line antimicrobial agents, with ciprofloxacin resistance exceeding 70% in *E. coli* isolates and ceftriaxone resistance affecting over 60% of isolates. These findings necessitate fundamental revisions to current prophylactic protocols and empirical treatment strategies. Contemporary guidelines should increasingly recommend local antibiogram-guided prophylaxis and consideration of broader-spectrum agents in high-risk patients or institutions with documented high resistance rates.

Early recognition of complications emerges as a critical clinical imperative from our imaging analysis, with intra-abdominal collections (51.2%) and pancreatitis (48.7%) representing the most common CT-detectable complications. The timing of most complications within 24-48 hours post-procedure underscores the importance of immediate post-procedural surveillance and rapid response protocols. Risk stratification based on identified patient and procedure factors can significantly improve patient selection processes and enhance informed consent discussions.

The findings from our included studies should inform clinical practice guidelines, quality improvement initiatives, and future research priorities in therapeutic endoscopy. Healthcare institutions and practitioners should consider implementing systematic surveillance protocols, evidence-based prophylactic strategies, and comprehensive complication management systems to optimize patient safety and outcomes in ERCP practice, considering the variability in findings from contemporary practice patterns identified in this review.

## Conclusions

This systematic review of contemporary ERCP practice demonstrates that complications remain a significant clinical challenge, with overall rates of 9.4-15.9% and mortality rates of 0.26-1.0%. PEP continues as the most common serious complication (3.8-17.2%), while infection-related complications show varied patterns of antimicrobial resistance, particularly high ciprofloxacin resistance (72.73% in *E. coli*) and reduced ceftriaxone susceptibility (37.50% in *E. coli*). The analysis reveals that contemporary ERCP procedures face evolving challenges, including changing microbial resistance patterns, complex patient comorbidities, and the need for individualized risk stratification. Key risk factors identified include advanced age, previous ERCP history, stent placement, and hilar obstruction, while protective factors include endoscopic stone extraction and experienced operator expertise. Based on the evidence quality from these 10 studies (two excellent, eight good quality), these findings suggest that contemporary ERCP procedures may benefit from updated antibiotic prophylaxis protocols, enhanced surveillance systems, and personalized risk management strategies. Further validation through large-scale multicenter trials is needed to guide evidence-based protocol development and optimize patient outcomes in contemporary ERCP practice.
